# Free Public Baths

**Published:** 1897-08

**Authors:** 


**A Monthly Review of Medicine and Surgery.**

*EDITORS:*

THOMAS LOTHROP, M. D. - - WM. WARREN POTTER, M. D.

All communications, whether of a literary or business nature, should be addressed to the managing editor: 284 FRANKLIN STREET, BUFFALO, N. Y.

VOL. XXXVII. AUGUST, 1897. No. 1.

### FREE PUBLIC BATHS.

THE great value of free public baths as a sanitary measure in large cities is already admitted. The success that has attended their establishment in Buffalo is somewhat phenomenal when considered in the light of novelty and prejudice. It was not a trifle to overcome the latter. Ignorance is always the stronghold of prejudice, and novelty serves as a feeder to opposition. At any rate, there are many people in the world and especially among the ignorant in the large cities, who do not brook improvement or welcome reform.

The free public bath system of Buffalo was opened January 1, 1897, and is reported to be the first of its kind in the United States. At all events, we propose to give a brief description of it and rest our claims to priority with the record. The visitor will find a substantial red brick structure standing on the upper terrace, two stories in front elevation, extending back a distance of about sixty feet. Entering the vestibule a turn to the left finds one in a comfortable office room, say 14 x 16, in which are found the desk of the superintendent, a stack of clean towels, a box of soap, neatly cut into small squares, fourteen chairs for the accommodation of those in waiting and a blackboard on the wall on which is taken the daily census of the bathers.

Entering the bath proper, first on the right is discovered an ordinary bathroom, fitted up with bathing tub and all the conveniences pertaining to a private bath, which is for the accommodation of feeble women and babies. Next adjoining is a water-closet, and then a pool room in which fifteen or twenty boys may be showered at one time. On the right and left of a long corridor are fourteen individual bathrooms, where each bather secures the privacy that belongs to an ordinary home bath. Receiving his towel and soap from the superintendent the bather goes directly through the corridor into the bathroom to which he is assigned, disrobes in the front section, thence passes into the bathroom proper, where he turns on hot and cold water to his liking. The bathers are received in groups of fourteen, and their places are immediately supplied in the waiting-room by fourteen others, who come from the crowd in waiting on the street. Twenty minutes are allowed to each group and they are required to take their turn in rapid succession.

The hours for men and boys are from 7 to 12 in the morning and for women and girls from 1 to 6 in the afternoon. About 500 people avail themselves of the privilege of the bath each day. We omitted to mention the fact that a laundry is provided where a bather may wash his shirt, hang it in the hot-air drying chamber, and it will be ready to put on when he emerges from the bath.

The influence of such a system on the health of the community must admittedly be considerable, and we know of no investment that yields so large a dividend in sanitary results. It is at the present season of the year especially important that frequent baths should be taken if health would be preserved, but experience has shown that the free public baths in this city are in nearly as great demand in the cold weather as in the warm season. Whoever may have conceived prejudice against the public baths by reason of supposed danger of infection, contagion or uncleanliness, has but to visit them and carefully inspect their whole management when all such prejudice will vanish.

In this relation it may not be out of place to contrast the Buffalo system with that in vogue in New York. At the Grand street free bath there is always a motley group waiting for admission. The baths are open from sunrise until late evening and they are occupied every moment of the day. In the late afternoon and evening they are specially besieged by hundreds who are pushing and fighting for a chance to plunge into the salt water which is rarely clean.

The bathers are admitted in crowds—in droves. At the most crowded hours there are sometimes in the largest of the baths several hundred bathers splashing and plunging and diving about. At times they are so numerous that they fairly obscure and fill the water. If the bathers would but hold their places, one could cross the water dry shod by stepping upon their heads. But they do everything but hold their places. The little dressing-rooms are packed like sardine boxes for about a half minute when a fresh relay of bathers is admitted. It takes no longer than that to undress and don the scanty trunks demanded, for time is precious in the baths.

There are no doors to the dressing-rooms, there are no keys, there is no place to deposit valuables. The east side brings no valuables to the baths. The east side knows the east side. It is lucky to find its clothes when the attendants chase it out of the water at the end of twenty minutes or so. Sometimes it doesn't find its clothes. Towels are unknown in the free baths. A man or boy who brought a towel to a free bath would at once mark himself as an aristocrat and would have a very bad quarter of an hour at the hands of his companions. Combs are eschewed with equal unanimity. All there is is water, and sometimes even that is of an inferior quality.

Buffalo may well be proud of her free public baths. Here the water is clean, hot or cold, or both ; towels and soap are furnished and the bather's clothes are safe while he is taking his ablutions. The baths are now permanently instituted, and for their successful management the city owes not a little to the health commissioner, Dr. E. Wende, who has from the first been a firm advocate of their establishment and has labored for their thorough sanitary conduct.

